# CD146 Deletion in the Nervous System Impairs Appetite, Locomotor Activity and Spatial Learning in Mice

**DOI:** 10.1371/journal.pone.0074124

**Published:** 2013-09-10

**Authors:** Tao Tu, Qian Gao, Yongting Luo, Jianan Chen, Di Lu, Jing Feng, Dongling Yang, Lina Song, Xiyun Yan

**Affiliations:** 1 Key Laboratory of Protein and Peptide Pharmaceuticals, CAS-University of Tokyo Joint Laboratory of Structural Virology and Immunology, Institute of Biophysics, Chinese Academy of Sciences, Beijing, China; 2 University of Chinese Academy of Sciences, Beijing, China; Alexander Flemming Biomedical Sciences Research Center, Greece

## Abstract

Cell adhesion molecules (CAMs) are crucial effectors for the development and maintenance of the nervous system. Mutations in human CAM genes are linked to brain disorders and psychological diseases, and CAM knockout mice always exhibit similar behavioral abnormalities. CD146 is a CAM of the immunoglobulin superfamily that interacts with Neurite Outgrowth Factor and involved in neurite extension *in vitro*. However, little is known about its *in vivo* function in the nervous system. In this study, we used a murine CD146 nervous system knockout (CD146^ns-ko^) model. We found that the brains of some CD146^ns-ko^ mice were malformed with small olfactory bulbs. CD146^ns-ko^ mice exhibited lower body weights and smaller food intake when compared with wild type littermates. Importantly, behavior tests revealed that CD146^ns-ko^ mice exhibited significant decreased locomotor activity and impaired capacity for spatial learning and memory. Our results demonstrate that CD146 is important for mammalian nervous system development and proper behavior patterns.

## Introduction

Cell adhesion molecules (CAMs) play an important role in the nervous system. They participate in every stage of neural development, facilitating neural stem cell proliferation and migration, neurite extension and path-finding, axon-axon fasciculation and synapse formation. CAMs are also required for structural maintenance and neural network regulation, as well as regeneration and neural repair in the adult nervous system [1]–[3]. More than merely molecular glue, CAMs bind to similar molecules (homophilic interactions) and non-similar molecules (heterophilic interactions), mediating outside-in signals to regulate cell functions [4], [5]. Mutations in human CAM genes such as neural cell adhesion molecule (NCAM), L1-CAM and close homologue of L1 (CHL1) are associated with brain disorders and psychological diseases such as schizophrenia and CRASH syndrome (Corpus callosum hypoplasia, Retardation, Adducted thumbs, Spastic paraplegia and Hydrocephalus) [6]–[8]. Disruption of rodent homologues always results in abnormalities both in the structure of the central nervous system (CNS) and in behavioral patterns, including decreased body weight, locomotor activity, impaired spatial learning, alternated anxiety levels, sociability and aggregation [9]–[12].

CD146 is a membrane CAM of the immunoglobulin (Ig) superfamily with high sequence similarity to NCAM and L1 [13]. This molecule was originally cloned from human melanoma cells and subsequently found expressed on various tumor cells [14]–[16]. In human normal tissues, CD146 is mainly expressed on the surface of endothelial cells and smooth muscle cells in various organs throughout the body, some subsets of leukocytes and certain categories of neuronal cells [17]. A similar expression pattern of CD146 has been reported in mice [18]. Previous studies have demonstrated that CD146 plays a critical role in angiogenesis and tumor metastasis [19]. However, its role in the nervous system remains to be elucidated. CD146 was identified as a Neurite Outgrowth Factor (NOF) binding protein, and has been shown to be involved in neurite extension *in vitro* [20]–[22]. Its chicken homologue, Gicerin, was found to be upregulated in the spinal cord following injury, suggesting that CD146 may play a role in neural regeneration [23]. Whilst CD146 is widely expressed in the nervous system, and involved in several neural processes [24], [25], there is no experimental data characterizing its *in vivo* role in the nervous system. It is not known, for instance, whether a deficiency in CD146 expression affects the development of the CNS and the behavior patterns of animals.

To study the potential function of CD146 in the mammalian nervous system, we constructed a murine CD146 nervous system knockout model, and assessed the health status, CNS development, and behavioral pattern of these mice.

## Materials and Methods

### Ethic Statement

All animal experiments were pre-reviewed and approved by the Biomedical Research Ethics Committee of the Institute of Biophysics, Chinese Academy of Sciences. The Biomedical Research Ethics Committee based its decision on “Regulations for the Administration of Affairs Concerning Experimental Animals” (approved by the State Council on October 31, 1988). The animal experiments were performed in compliance with the Guidelines for the Care and Use of Laboratory Animals (Ministry of Science and Technology, NO. 398, 2006).

### Animal Studies

Nes^cre/+^CD146^floxed/floxed^ mice were generated using a Cre/loxP recombination system [26]. Briefly, Nes^+/+^CD146^floxed/floxed^ mice were generated by inserting two loxP sites into the promoter and the 1^st^ intron of the CD146 gene. Then the mice were crossed to a C57BL/6J background for a minimum of nine generations. The C57BL/6J background Nes^+/+^CD146^floxed/floxed^ mice were then mated with B6.Cg(SJL)-TgN(NesCre)1Kln mice (Nes^cre/+^CD146^+/+^) purchased from Jackson laboratories (Maine, USA). The F1 Nes^cre/+^CD146^floxed/+^ genotype was back-crossed with Nes^+/+^CD146^floxed/floxed^ mice to obtain Nes^cre/+^CD146^floxed/floxed^ (CD146^ns-ko^) mice. Nes^+/+^CD146^floxed/floxed^ (WT) mice were used as controls. Genotyping of each generation was carried out using tail-PCR. Immunohistochemistry and RT-PCR assays were used to confirm CD146 deficiency in the nervous system.

All the experimental mice were housed in Individually Ventilated Cages with standard cage bedding (Aspen chips) and had no more than five cage companions. During the experiments, mice were housed under specific-pathogen-free conditions with 12-hour light/dark cycle and controlled temperature (20°C to 25°C) and fed normal chow and water ad libitum at Laboratory Animal Center of Institute of Biophysics, Chinese Academy of Sciences (Beijing, China).

### Histology and Immunohistochemistry (IHC)

To study the expression pattern of CD146 in mouse CNS and evaluate the knockout efficiency, WT and CD146^ns-ko^ mice at the age of 3 months were sacrificed by cervical dislocation performed by well-trained individuals. Brains and spinal cords were carefully dissected and then fixed with 4% paraformaldehyde in phosphate buffered saline (PBS, pH 7.4). Following storage for 2–3 days, the tissues were embedded in paraffin and sectioned at 5 µm. Sagittal and coronal cuts were used for brain sectioning. An anti-CD146 monoclonal antibody AA4 generated in our lab [27] was used as the primary antibody followed by Biotin-conjugated anti-mouse secondary antibody (ZSGB-BIO, Beijing, China) and horseradish peroxidase-conjugated streptavidin (Thermo Fisher, Massachusetts, USA). The DAB substrate and Harris haematoxylin used for staining were purchased from ZSGB-BIO (Beijing, China).

For hippocampal area and neuron density measurement, 1-month-old male WT and CD146^ns-ko^ mice (n = 3 each phenotype) were sacrificed after the Morris water maze test. Left hemispheres of the brains were sagittally sectioned at 5 µm with 200 µm intervals and then Nissl staining was performed. Five sections from each brain that approximate 200, 400, 600, 800 and 1000 µm apart from the midline were used for hippocampal area measurement, and the sections 200 µm apart from the midline were used for neuron density analysis. Hippocampal area and neuron density of CA1, CA3 and dentate gyrus (DG) areas were quantified with NIH ImageJ.

### RT-PCR

To verify that CD146 expression in the nervous system of CD146^ns-ko^ mice was disrupted, total mRNA from whole brains, cerebral cortexes and spinal cords of 2 WT and 4 CD146^ns-ko^ adult mice at the age of 3 months was isolated using Trizol reagent (Life Technologies, California, USA). mRNA was then reverse-transcribed into cDNA according to the manufacturer’s instructions (Life Technologies, California, USA). CD146 mRNA expression was measured using the following primers for mCD146, and was normalized against mouse GAPDH (mGAPDH).

CD146 primer F: 5′-aggaccttgagtttgagtgg-3′

CD146 primer R: 5′-cagtggtttggctggagt-3′

### Determination of Body Weight and Food Intake

For body weight measurement, WT and CD146^ns-ko^ mice at the ages of 1 month, 3 months and 6 months of both male (n = 7, 7, 7 WT mice at the ages of 1 month, 3 months and 6 months respectively. n = 10, 8, 7 CD146^ns-ko^ mice at the ages of 1 month, 3 months and 6 months respectively) and female (n = 6, 5, 6 WT mice at the ages of 1 month, 3 months and 6 months respectively. n = 7, 7, 6 CD146^ns-ko^ mice at the ages of 1 month, 3 months and 6 months respectively) were weighted in the house room. Then the mice at the age of 1 month were subjected to daily food intake measurement. Briefly, mice were separated and placed in cages at 10∶00 am, before which food was weighed. At the same time the following day, the remaining food was weighed again. Careful inspection was carried out to ensure no food was hidden. The difference in weight of the food represented food consumption over the 24-hour period. The experiment was performed for 4 weeks with food intake being measured on the same day each week.

### The Rotarod Test and the Open Field Test

Motor coordination and balance were measured using the Rotarod apparatus (Panlab, Barcelona, Spain) installed in the house room before the animals arrived. In the Rotarod test, mice at the ages of 1 month and 3 months were used (n = 13 and 12 WT mice at the ages of 1 month and 3 months respectively. n = 17 and 15 CD146^ns-ko^ mice at the ages of 1 month and 3 months respectively). Briefly, mice were placed on an accelerating 3-cm diameter cylinder. The initial rotation speed of 10 rpm was gradually increased to 40 rpm over a 20-second period. Latency to fall was recorded automatically for each mouse with a 30 s cut-off time in three consecutive trials.

The open field test was performed to assess general locomotor activity. In the open field test, mice at the ages of 1 month, 3 months and 6 months were used (n = 13, 12, 13 WT mice at the ages of 1 month, 3 months and 6 months respectively. n = 17, 15, 13 CD146^ns-ko^ mice at the ages of 1 month, 3 months and 6 months respectively). The open field was prepared using a white plastic tray 40 cm long, 40 cm wide and 30 cm deep, with the central area being defined as 20 cm×20 cm area in the center of the tray. Each mouse was transferred to the same corner of the chamber, and then a 5-min testing session was performed. The distance travelled, rest time, maximum speed and time spent in the central area were recorded by a smart video tracking system (Panlab, Barcelona, Spain). Tests were performed between 10∶00 am and 12∶00 am under standard room light conditions. Chambers were cleaned with 70% ethanol between each test.

### The Morris Water Maze Test

The spatial learning and memory capacity of mice was assessed in the Morris water maze (n = 11 WT and n = 12 CD146^ns-ko^ mice). Briefly, a circular pool with high-contrast geometrical patterns mounted on the wall was used. The water was mixed with nontoxic white paint and maintained at 25°C. A hidden 10-cm diameter platform made of clear Plexiglas was prepared. The upper surface of the platform was 1 cm below the surface of water to ensure it was invisible to the mice. On the first day, the mice were placed on the platform for 60 s for pre-training. Then during training (day 2 to 8), trials were performed by lowering the animal into the water with its head facing the wall at one of three starting positions (chosen randomly), each located in a different quadrant of the pool but not in the target quadrant. Animals were given 90 s to find the target and the time taken to find the platform was recorded. If the mouse did not find the platform within 90 s, it was manually guided to the platform to rest on it for 15 s. On the day after training, the platform was removed and the mice were allowed to swim freely for 5 min. Time spent in each quadrant was recorded. During the experiment, all trials were recorded using a smart video tracking system (Panlab, Barcelona, Spain).

### Statistical Analysis

The data followed a normal distribution, and are expressed as the mean ± standard error of the mean (SEM). A chi-square test was performed to compare the incidence of abnormal olfactory bulbs in WT and CD146^ns-ko^ groups. Two-way ANOVA was used to analyze the data of body weight, Rotarod test and open field test. For food intake, Morris water maze training test and hippocampal area measurement, analysis was performed using a repeated measures ANOVA. For Morris water maze probe test and neuron density evaluation, analysis was performed using Student’s t test. The criterion for statistical significance was defined as p<0.05.

## Results

### 1. Generation and Identification of CD146^ns-ko^ Mice

**Figure 1:**
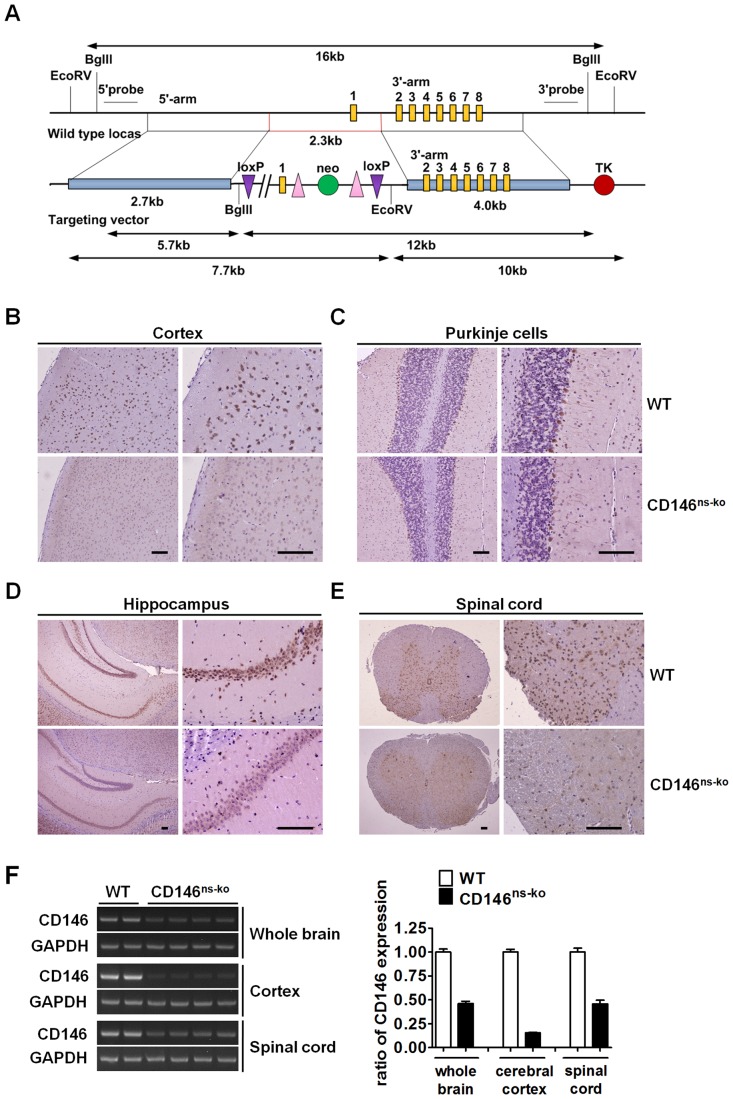
Generation and identification of CD146^ns-ko^ mice. (A) Schematic strategy for constructing CD146^floxed/floxed^ mice. Two loxP sites were inserted into the promoter and 1^st^ intron of the CD146 gene by targeting vector. (B–E) Sections of brains and spinal cords of WT and CD146^ns-ko^ mice were immunostained with anti-CD146 mAb AA4. Expression of CD146 was observed on cortical neurons (B), cerebellar Purkinje cells (C), hippocampal neurons (D) in brain and neurons in spinal cord (E). Scale bar represents 200 µm. (F) Expression of CD146 in whole brain, cerebral cortex alone and spinal cord of WT and CD146^ns-ko^ mice was analyzed in cDNA RT-PCR assay. Data is presented as ratio of CD146 expression level normalized with WT group (means ± SEM).

To study the potential function of CD146 in the vertebrate nervous system, we generated CD146^ns-ko^ mice by mating CD146^floxed/floxed^ mice with Nes^cre/+^ mice. LoxP sites were inserted into the CD146 gene as shown in Figure 1A. Absence of CD146 expression in the nervous system of CD146^ns-ko^ mice was verified by immunohistochemistry (IHC) and cDNA RT-PCR. Immunostaining indicated that CD146 was mainly expressed on the surface of cortical neurons (Figure 1B), cerebellar Purkinje cells (Figure 1C), and hippocampal neurons (Figure 1D) in the brain and neurons in the spinal cord (Figure 1E). In contrast, expression levels were below detection limits in CD146^ns-ko^ mice, except for some weak staining on the surface of Purkinje cells. The expression pattern of CD146 in the adult mouse CNS partly resembles that in the rat, with staining of hippocampus neurons and Purkinje cells being observed. However, strong CD146 staining was observed in the mouse cerebral cortex, but has not been reported yet for rat [24]. CD146 mRNA levels in whole brain tissue samples and spinal cord samples of CD146^ns-ko^ mice, as measured by RT-PCR, were less than half that of WT mice. Moreover, this ratio was even lower when cerebral cortex samples alone were compared (Figure 1F). Given that we cannot exclude the involvement of CD146-positive endothelial and other supporting cells in RT-PCR assay, our results together suggest that CD146 surface protein expression on neural cells was successfully abolished.

### 2. CD146^ns-ko^ Mice Show Stochastic Reduction in the Size of Olfactory Bulbs

**Figure 2:**
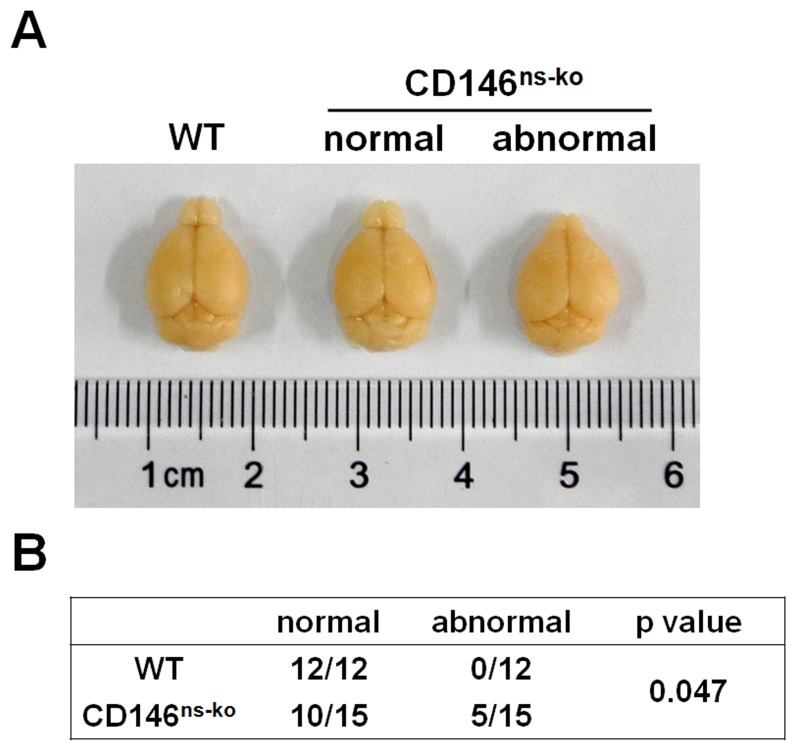
CD146^ns-ko^ mice show stochastic reduction in the size of olfactory bulbs. (A) Representative images of WT brain, CD146^ns-ko^ normal brain and CD146^ns-ko^ abnormal brain. (B) A Chi-square test was performed to compare the incidence of abnormal olfactory bulbs in WT and CD146^ns-ko^ groups.

We next investigated whether CD146 neural knockout has an effect on the morphogenesis of the mouse brain. The total weight of adult brains was measured, and no difference was found between CD146^ns-ko^ mice and WT mice (data not shown). However, a proportion of CD146^ns-ko^ mice (5/15) exhibited pointy olfactory bulbs that were greatly reduced in size, clearly an abnormality that could not be explained simply by natural size variation (Figure 2A), which was not observed in WT group. A chi-square test was performed and the results showed that the incidence of abnormal olfactory bulbs is significantly higher in CD146^ns-ko^ group than in WT group (Figure 2B). This result suggests that CD146 can affect normal development of the olfactory bulb in mice, possibly in a random manner.

### 3. Knockout of CD146 in the Nervous System Results in Decreased Body Weight and Food Intake in Mice

**Figure 3:**
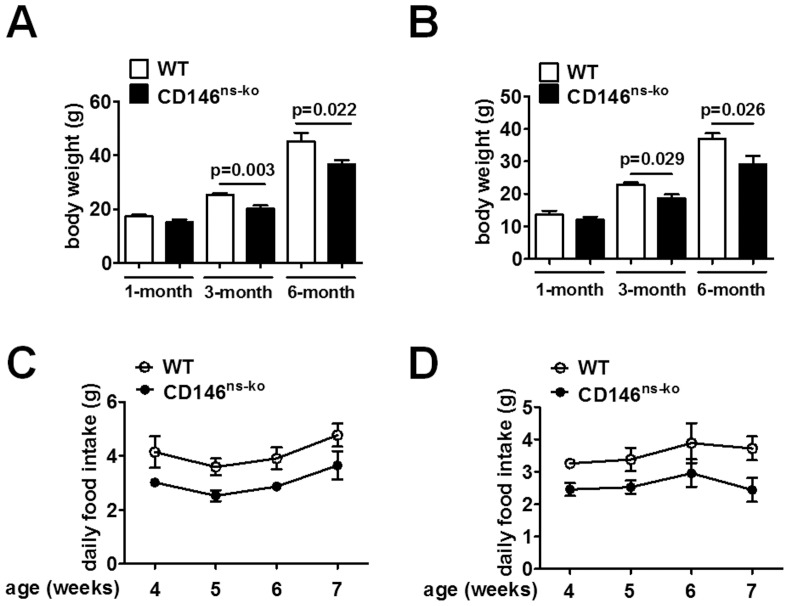
Knockout of CD146 in the nervous system resulted in decreased body weight and food intake in mice. Body weight of male (A) and female (B) mice at the ages of 1 month, 3 months and 6 months were measured and analyzed with a two-way ANOVA. Daily food intake of male (C) and female (D) mice at the age of 1 month was measured once a week for 4 weeks. Data is presented as means ± SEM and analyzed by a repeated measures ANOVA.

We next checked the overall health status of CD146^ns-ko^ mice. These mice were viable, fertile and appeared normal during adolescence. However, several weeks after weaning, CD146^ns-ko^ mice were smaller than WT mice. We measured the body weight of the mice at the ages of 1 month, 3 months and 6 months, and analyzed the data with a two-way ANOVA for genotype and age. Significant differences were found between these two genotypes in both male (F_1,40_ = 18.282, p<0.001; Figure 3A) and female (F_1,31_ = 13.994, p = 0.001; Figure 3B) mice. Post-hoc tests showed that a significant difference was not detected between mice at 1 month old, and weight differences were evident at the ages of 3 and 6 months. Since the difference in body weight only appeared in mice older than 1 month, we tested the daily food intake of CD146^ns-ko^ and WT mice from the age of 1 month to around 2 months. A repeated measures ANOVA was performed in analysis of daily food consumption, and the results showed that both male (F_1,11_ = 8.101, p = 0.016; Figure 3C) and female (F_1,11_ = 7.240, p = 0.021; Figure 3D) CD146^ns-ko^ mice consumed less than their WT littermates. The decreased food consuming in CD146^ns-ko^ mice probably resulted in the observed differences in body weight.

### 4. Knockout of CD146 in the Nervous System Results in Decreased Locomotor Activity in Mice

**Figure 4:**
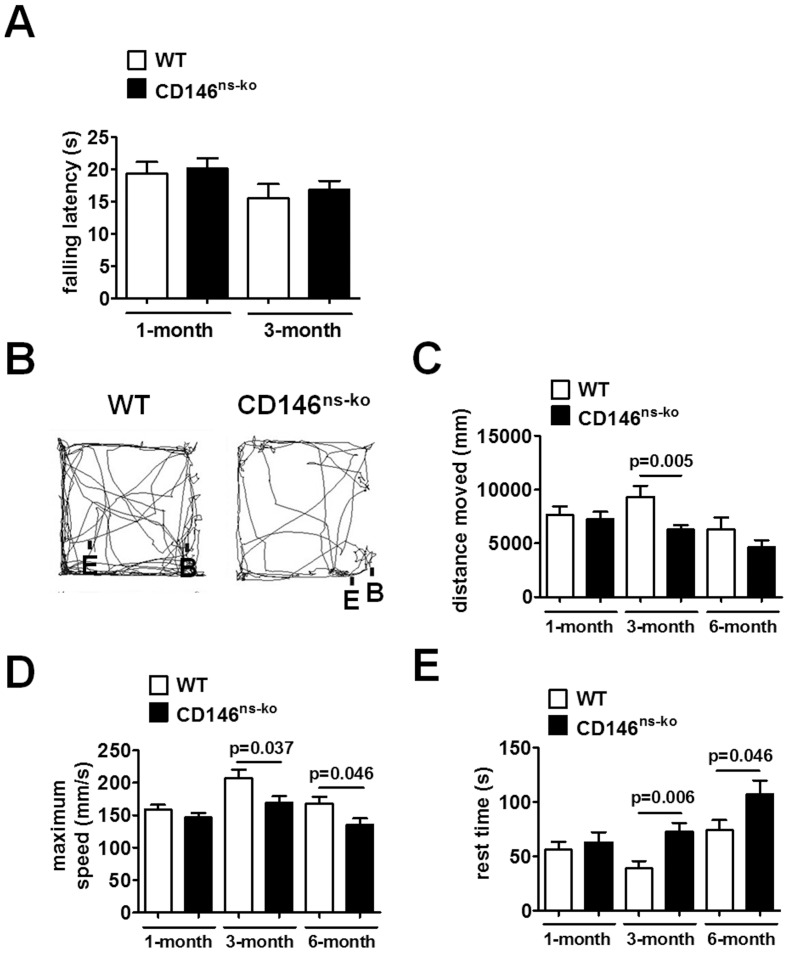
Knockout of CD146 in the nervous system resulted in decreased locomotor activity in mice. (A) Mice at the ages of 1 month and 3 months were subjected to the Rotarod test. Falling latency was recorded with a 30 seconds cut-off time. Mice at the ages of 1 month, 3 months and 6 months were subjected to the open field test. Representative running tracks of mice at the age of 3 months were shown (B). B: begin-point. E: end-point. During the 5-min free running, distance travelled (C), maximum speed (D) and rest time (E) of WT and CD146^ns-ko^ mice were recorded and analyzed with a two-way ANOVA. Data is presented as means ± SEM.

Since CD146^ns-ko^ mice appeared to be more quiescent than WT mice, we looked for changes in motor functions as a result of CD146 deficiency. CD146^ns-ko^ mice performed normally in the Rotarod test (F_1,53_ = 0.351, p = 0.556; Figure 4A), indicating normal motor coordination and balance. However, during free running in the open field test, CD146^ns-ko^ mice travelled shorter distances (F_1,77_ = 7.078, p = 0.009; Figure 4C), at slower maximum speeds (F_1,77_ = 10.856, p = 0.001; Figure 4D), and rested longer (F_1,77_ = 10.400, p = 0.002; Figure 4E) than WT mice. Post-hoc tests showed that while 1-month-old mice behaved normally, 3- and 6-month-old CD146^ns-ko^ mice showed significant decreased locomotor activity than WT littermates. The representative tracts of WT CD146^ns-ko^ mice at the age of 3 months were shown in Figure 4B. The reduction in locomotor activity suggests that CD146 expression in the CNS is important for normal locomotor behavior. In addition, we did not observe any significant differences in the time spent in the central area of the open field, a measure taken as an indication of anxiety status (data not shown).

### 5. CD146 Deletion in the Nervous System Impairs Spatial Learning and Memory in Mice

**Figure 5:**
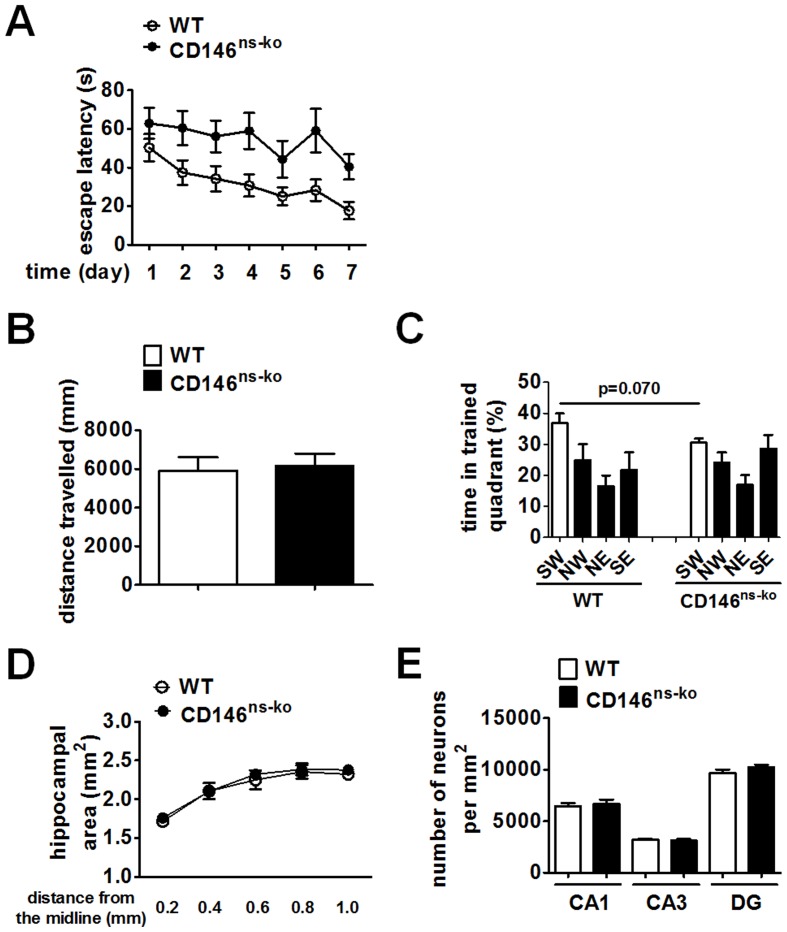
Knockout of CD146 in the nervous system impaired spatial learning and memory in mice. (A) WT and CD146^ns-ko^ mice at the age of 1 month were subjected to the Morris water maze test. During the 7-day training period, time spent in finding the target of each mouse was recorded and analyzed with a repeated measures ANOVA. At the last day of probe test, distance swam (B) and time spent in each quadrant by the mice (C) were recorded and analyzed with Student’s t test. The brains of 1-month-old WT and CD146^ns-ko^ mice were sagittally sectioned. The hippocampal area (D) and neuron density of CA1, CA3 and DG (E) were measured after Nissl staining. Data is presented as means ± SEM.

Mutations in CAM genes have been reported to be connected with deficiency in spatial learning and memory [9], [28], [29]. We then tested whether CD146 deletion has an effect on this trait using the Morris water maze. This test was performed on mice at the age of 1 month, to avoid potential influence from the decreased locomotor activity observed in older adult CD146^ns-ko^ mice. During the 7-day training period, the latency progressively decreased with days (F_6,126_ = 5.158, p<0.001), suggesting that all the mice were able to learn the task. However, the CD146^ns-ko^ mice spent markedly more time to find the target than WT mice (F_1,21_ = 9.066, p = 0.007; Figure 5A), indicating that CD146 nervous system knockout caused spatial learning defects. On the last day, the platform was removed, and mice were subjected to a 5-min interval of free swimming. There was no significant difference in swimming distance between CD146^ns-ko^ mice and WT mice (p = 0.8019, Figure 5B), confirming unchanged locomotor activity in 1-month-old CD146^ns-ko^ mice. Both groups spent more than 25% of the time in the target quadrant (p = 0.005 for CD146^ns-ko^ group and p = 0.008 for WT group). Moreover, though the statistical analysis indicates that the average times spent in the target quadrant by WT and CD146^ns-ko^ mice were not significantly different, we did observe a trend that CD146^ns-ko^ mice searched the target for a less time than their WT littermates (Figure 5C). Collectively, these results show that deletion of CD146 in the nervous system results in impaired spatial learning and memory in mice.

Since the hippocampus has been shown closely related to the spatial learning and memory in vertebrates [30], [31], we then checked whether CD146 deletion caused structural abnormalities in hippocampus. The hippocampal area (F_1,4_ = 0.820, p = 0.416; Figure 5D) and neuron density of CA1, CA3 and DG areas (Figure 5E) of WT and CD146^ns-ko^ brains were measured yet no significant differences were found between these two groups. These results suggest that CD146 deletion did not cause severe malformation of the hippocampus, but impaired spatial learning and memory ability in mice.

## Discussion

The function of CD146 in the development and maintenance of the CNS has not been studied in great detail, despite its wide distribution in the CNS and its involvement in several neural processes. Whilst previous studies have investigated the function of CD146 *in vitro* and *ex vivo,* here, we provide the first *in vivo* evidence for a crucial role of CD146 in mammalian nervous system development and maintenance. Using a CD146 tissue-specific knockout mouse system, we showed that lack of CD146 expression in the nervous system resulted in specific and significant physical and psychological deficiencies. First, CD146^ns-ko^ mice exhibited reduced food intake and were smaller compared with WT littermates. Second, in the open field test, locomotor activity in CD146^ns-ko^ mice was markedly decreased. Third, spatial learning and memory capacity was impaired in these mice in the Morris water maze test. Interestingly, these deficiencies did not occur simultaneously, but at different stages of life, showing that CD146 is crucial both in the early development and maintenance of the nervous system in the adult mouse. Moreover, the deficiencies observed were in highly specific processes and did not result from a broader impairment of the CNS, as indicated by results of the Rotarod test (Figure 4A), in which CD146^ns-ko^ mice exhibited normal balance comparable with WT litter mates.

Deletion of CD146 in the nervous system of mice disrupted appetite and food consumption, the only path of energy intake in animals. To our knowledge, this deficiency has not been reported in other CAM knockout mice. Appetite regulation is a poorly understood and complex process involving the gastrointestinal tract, hormones and the nervous system [32], [33]. Disrupted appetite causes anorexia and malnutrition in humans. Our results provide new insights into appetite regulation, suggesting that CAMs may take part in this process.

In the mammalian brain, the hippocampus is the most important area that responsible for learning and memory. Intriguingly, hippocampal neurons show strong CD146 surface expression, yet we were unable to identify any structural abnormalities in this area in mice lacking CD146. Even though the exact molecular mechanisms remain unclear, we reason that a compensatory mechanism during CNS development might be responsible for the partial restoration of the lack of CD146 in our CD146^ns-ko^ mice, resulting in little or no structure change but memory impairment. Unexpectedly, some CD146^ns-ko^ mice had very small olfactory bulbs, while CD146 expression was not detectable in olfactory bulbs in adult mice. We propose that, similar to NCAM [34], [35], CD146 might be expressed on neural stem cells during the early stages of development and facilitates the migration of these cells to the olfactory bulb. Thus elimination of CD146 affected neural stem cell migration and resulted in not fully developed olfactory bulbs. Interestingly, this only happened in a small proportion of mice, suggesting an auxiliary function for CD146 and the existence of redundant mechanisms, thus giving rise to a random effect, rather than being deterministic.

CAMs are known to be of particular importance during the development of the nervous system. For instance, NCAM and L1 participate in neuron survival and migration as well as axon growth, fasciculation and path-finding [36]–[39], and knockout of these molecules in rodents leads to a malfunction of the nervous system. The phenotype of CD146^ns-ko^ mice resembles that of NCAM and L1 knockout mice. NCAM knockout mice have small olfactory bulbs and defective spatial learning, however, no decrease in their locomotor activity has been observed [9]. In contrast, decreased body weight, defective locomotor activity and spatial learning have been observed in L1 knockout mice with no abnormalities in olfactory bulb development [10], [28]. Since both the gene sequences and knockout phenotypes in these mice are similar, we propose that CD146 plays a similar role to NCAM and L1, possibly resulting in redundancies in signaling pathways that are important for nervous system development. Our study not only demonstrates an important role of CD146, but also provides further proof that CAMs are essential in the development of the nervous system. Moreover, inactivation of CAMs in the nervous system of rodents resembles some gene mutations observed in humans which are linked to brain disorders and psychological diseases [12]. The possibility of an involvement of CD146 mutations in human psychological diseases such as anorexia and mental retardation warrants further investigation of this essential neuronal cell surface CAM.

## Supporting Information

Table S1 The Animal Research: Reporting In Vivo Experiments (ARRIVE) checklist.
(DOC)
