# Supplementary material for: CD146 Deletion in the Nervous System Impairs Appetite, Locomotor Activity and Spatial Learning in Mice
Source: PLoS One. 2013 Sep 10;8(9):e74124. doi: 10.1371/journal.pone.0074124 (PMC3769362; doi:10.1371/journal.pone.0074124)
Supplement: Table S1 — The Animal Research: Reporting In Vivo Experiments (ARRIVE) checklist. (DOC) [file pone.0074124.s001.doc]

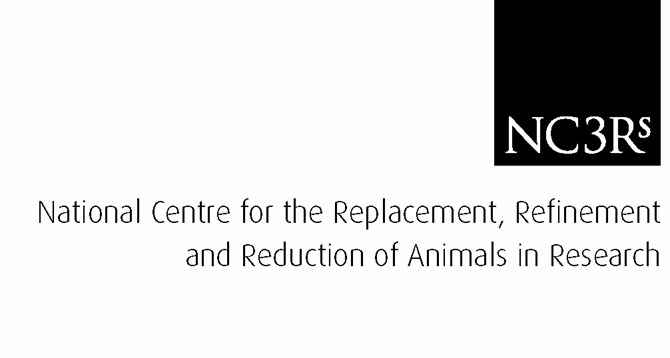


The ARRIVE guidelines

Animal Research: Reporting *In Vivo* Experiments

|  | **ITEM** | **RECOMMENDATION** | **REPORTED ON PAGE** |
| --- | --- | --- | --- |
| **TITLE** | 1 | CD146 Deletion in the Nervous System Impairs Appetite, Locomotor Activity and Spatial Learning in Mice | 1 |
| **ABSTRACT** | 2 | Cell adhesion molecules (CAMs) are crucial effectors for the development and maintenance of the nervous system. Mutations in human CAM genes are linked to brain disorders and psychological diseases, and CAM knockout mice always exhibit similar behavioral abnormalities. CD146 is a CAM of the immunoglobulin superfamily that interacts with Neurite Outgrowth Factor and involved in neurite extension *in vitro*. However, little is known about its *in vivo* function in the nervous system. In this study, we used a murine CD146 nervous system knockout (CD146^ns-ko^) model. We found that the brains of some CD146^ns-ko^ mice were malformed with small olfactory bulbs. CD146^ns-ko^ mice exhibited lower body weights and smaller food intake when compared with wild type littermates. Importantly, behavior tests revealed that CD146^ns-ko^ mice exhibited significant decreased locomotor activity and impaired capacity for spatial learning and memory. Our results demonstrate that CD146 is essential for mammalian nervous system development and proper behavior patterns. | 2 |
| **INTRODUCTION** | | |  |
| **Background** | 3 | Cell adhesion molecules (CAMs) play an important role in the nervous system. They participate in every stage of neural development, facilitating neural stem cell proliferation and migration, neurite extension and path-finding, axon-axon fasciculation and synapse formation. CAMs are also required for structural maintenance and neural network regulation, as well as regeneration and neural repair in the adult nervous system. More than merely molecular glue, CAMs bind to similar molecules (homophilic interactions) and non-similar molecules (heterophilic interactions), mediating outside-in signals to regulate cell functions. Mutations in human CAM genes such as NCAM, L1 and CHL1 are associated with brain malformation and psychological diseases such as schizophrenia and CRASH syndrome. Disruption of rodent homologues always results in abnormalities both in the structure of the central nervous system (CNS) and in the behavioral patterns.  CD146 is a membrane CAM of the immunoglobulin (Ig) superfamily with high sequence similarity to NCAM and L1. This molecule was originally cloned from human melanoma cells and subsequently found expressed on various tumor cells. In normal tissues, CD146 is mainly expressed on the surface of endothelial cells, smooth muscle cells, some subset of leukocytes and certain categories of neuronal cells. Previous studies have demonstrated that CD146 plays a critical role in angiogenesis and tumor metastasis. However, its role in the nervous system remains to be elucidated. CD146 was identified as a Neurite Outgrowth Factor (NOF) binding protein, and has been shown to be involved in neurite extension *in vitro*. Its chicken homologue, Gicerin, was found to be upregulated in the spinal cord following injury, suggesting that CD146 may play a role in neural regeneration. Whilst CD146 is widely expressed in the nervous system, and involved in several neural processes, there is no experimental data characterizing its *in vivo* role in the nervous system. | 2-3 |
| **Objectives** | 4 | To investigate the *in vivo* role of CD146 in nervous system development and maintenance, we generated a mouse strain lacking CD146 in the nervous system (named CD146^ns-ko^) using Cre/loxp recombinant system. Structure analysis of the central nervous system in CD146^ns-ko^ mice revealed abnormal development of the brain with small olfactory bulbs. Food intake was reduced and body size was significantly smaller in CD146^ns-ko^ mice compared with wild type (WT) littermates. Importantly, locomotor activity and spatial learning were impaired markedly in these mice. Collectively, our findings demonstrate that CD146 is indispensable for the proper development and maintenance of the mammalian nervous system. | 3-4 |
| **METHODS** | | |  |
| **Ethical statement** | 5 | All animal experiments were pre-reviewed and approved by the Biomedical Research Ethics Committee of the Institute of Biophysics, Chinese Academy of Sciences. The Biomedical Research Ethics Committee based its decision on “Regulations for the Administration of Affairs Concerning Experimental Animals” (approved by the State Council on October 31, 1988). The animal experiments were performed in compliance with the Guidelines for the Care and Use of Laboratory Animals (Ministry of Science and Technology, NO.398, 2006). | 4 |
| **Study design** | 6 | In each experiment, CD146^ns-ko^ mice and WT mice (as controls) in C57BL/6J background were used. The body weight of mice at the ages of 1 month, 3 months and 6 months were measured. Then the 1-month and 3-month old mice were subjected to the Rotarod test and all the mice were subjected to the open field test. After that, the 3-month-old mice were sacrificed to investigate whether CD146 deletion caused any structural abnormity of the brain, and the 1-month-old mice were subjected to daily food intake measurement. The Morris water maze test was conducted with separate, unhandled groups of 1-month-old mice. For the body weight and food intake measurements, male mice and female mice were allocated to separate groups while in other tests, they were not differentiated since no internal differences in their performance were found between two genders. | - |
| **Experimental procedures** | 7 | Structure analysis of the brain  WT and CD146^ns-ko^ mice at the age of 3 months were sacrificed by cervical dislocation performed by well-trained individuals. The brains were carefully dissected and then fixed with 4% paraformaldehyde in phosphate buffered saline (PBS, pH 7.4). The brains were then photographed and the length of each olfactory bulb was measured.  Determination of body weight and food intake  For body weight measurement, WT and CD146^ns-ko^ mice at the ages of 1 month, 3 months and 6 months of both male and female were weighted in the house room. Then the mice at the age of 1 month were subjected to daily food intake measurement. Briefly, mice were separated and placed in cages at 10:00 am, before which food was weighed. At the same time the following day, the remaining food was weighed again. Careful inspection was carried out to ensure no food was hidden. The difference in weight of the food represented food consumption over the 24 h period. The experiment was performed for 4 weeks with food intake being measured on the same day each week.  The Rotarod test and the open field test  Motor coordination and balance were measured using the Rotarod apparatus (Panlab) installed in the house room before the animals arrived. In the Rotarod test, mice at the ages of 1 month and 3 months were used. Briefly, mice were placed on an accelerating 3-cm diameter cylinder. The initial rotation speed of 10 rpm was gradually increased to 40 rpm over a 20-second period. Latency to fall was recorded automatically for each mouse with a 30 s cut-off time in three consecutive trials.  The open field test was performed to assess general locomotor activity. In the open field test, mice at the ages of 1 month, 3 months and 6 months were used. The open field was prepared using a white plastic tray 40 cm long, 40 cm wide and 30 cm deep, with the central area being defined as 20 cm × 20 cm area in the center of the tray and installed in the house room before the animals arrived. Each mouse was transferred to the same corner of the chamber, and then a 5-min testing session was performed. The distance travelled, rest time, maximum speed and time spent in the central area were recorded by a smart video tracking system (Panlab). Tests were performed between 10:00 am and 12:00 am under standard room light conditions. Chambers were cleaned with 70% ethanol between each test.  The Morris water maze test  The spatial learning and memory capacity of mice was assessed in the Morris water maze. Briefly, a circular pool with high-contrast geometrical patterns mounted on the wall was used. The water was mixed with nontoxic white paint and maintained at 25°C. A hidden 10-cm diameter platform made of clear Plexiglas was prepared. The upper surface of the platform was 1 cm below the surface of water to ensure it was invisible to the mice. On the first day, the mice were placed on the platform for 60 s for pre-training. Then during training (day 2 to 8), trials were performed by lowering the animal into the water with its head facing the wall at one of three starting positions (chosen randomly), each located in a different quadrant of the pool but not in the target quadrant. Animals were given 90 s to find the target and the time taken to find the platform was recorded. If the mouse did not find the platform within 90 s, it was manually guided to the platform to rest on it for 15 s. On the day after training, the platform was removed and the mice were allowed to swim freely for 5 min. Time spent in each quadrant was recorded. Experiments were performed between 10:00 am and 12:00 am and all trials were recorded using a smart video tracking system (Panlab). | 5-8 |
| **Experimental animals** | 8 | Nes^cre/+^CD146^floxed/floxed^ mice were generated using a Cre/loxP recombination system. Briefly, Nes^+/+^CD146^floxed/floxed^ mice were generated by inserting two loxp sites into the promoter and the 1^st^ intron of the CD146 gene. Then the mice were crossed to a C57BL/6J background for a minimum of nine generations. The C57BL/6J background Nes^+/+^CD146^floxed/floxed^ mice were then mated with B6.Cg(SJL)-TgN(NesCre)1Kln mice (Nes^cre/+^CD146^+/+^) purchased from Jackson laboratories. The F1 Nes^cre/+^CD146^floxed/+^ genotype was back-crossed with Nes^+/+^CD146^floxed/floxed^ mice to obtain Nes^cre/+^CD146^floxed/floxed^ (CD146^ns-ko^) mice. Nes^+/+^CD146^floxed/floxed^ (WT) mice were used as controls. Genotyping of each generation was carried out using tail-PCR. CD146^ns-ko^ and WT mice at the ages of 1 month, 3 months and 6 months of both genders were used in the behaviour experiments. | 5 |
|  | **ITEM** | **RECOMMENDATION** | **REPORTED ON PAGE** |
| **Housing and husbandry** | 9 | All the experimental mice were housed in Individually Ventilated Cages with standard cage bedding (Aspen chips) and had no more than five cage companions. During the experiments, mice were housed under specific-pathogen-free conditions with 12-hour light/dark cycle and controlled temperature (20°C to 25°C) and fed normal chow and water ad libitum at Laboratory Animal Center of Institute of Biophysics, Chinese Academy of Sciences. | 5 |
| **Sample size** | 10 | For measuring the length of olfactory bulbs, a total number of 27 brains from mice at the age of 3 months were used. N=12 of WT mice and N=15 of CD146^ns-ko^ mice.  In body weight measurement, a total number of 83 mice were used. N=7, 7, 7 of male WT mice at the ages of 1 month, 3 months and 6 months respectively. N=10, 8, 7 of male CD146^ns-ko^ mice at the ages of 1 month, 3 months and 6 months respectively. N=6, 5, 6 of female WT mice at the ages of 1 month, 3 months and 6 months respectively. N=7, 7, 6 of female CD146^ns-ko^ mice at the ages of 1 month, 3 months and 6 months respectively.  In food intake measurement, a total number of 30 mice at the age of 1 month were used. N=7 of male WT mice, N=10 of male CD146^ns-ko^ mice. N=6 of female WT mice, and N=7 of female CD146^ns-ko^ mice.  In the Rotarod test, a total number of 57 mice were used. N=13 and 12 of WT mice at the ages of 1 month and 3 months respectively. N=17 and 15 of CD146^ns-ko^ mice at the ages of 1 month and 3 months respectively.  In the open field test, a total number of 83 mice were used. N=13, 12, 13 of WT mice at the ages of 1 month, 3 months and 6 months respectively. N=17, 15, 13 of CD146^ns-ko^ mice at the ages of 1 month, 3 months and 6 months respectively.  In the Morris water maze test, a total number of 23 mice at the age of 1 month were used. N=11 of WT mice and N=12 of CD146^ns-ko^ mice. | - |
| **Allocating animals to experimental groups** | 11 | The mice were allocated to experimental groups according to their genotypes (Nes^cre/+^CD146^floxed/floxed^ or Nes^+/+^CD146^floxed/floxed^), ages (1-month, 3-month or 6-month old) and genders (male or female). In each behavior experiment, the WT and CD146^ns-ko^ mice were assessed in a randomized order. | - |
| **Experimental outcomes** | 12 | To investigate whether CD146 deletion causes structure abnormality of the brain, the length of olfactory bulb of each brain was measured and analyzed.  For body weight determination, the body weight of each mouse was measured and analyzed.  For food intake measurement, the food consumption of each mouse in 24 h were recorded and analyzed.  In the Rotarod test, the average fall latency of each mouse in three consecutive tests were recorded and analyzed, with a 30 s cut-off time.  In the open field test, the length travelled, rest time and maximum speed of each mouse during a 5-min free running were recorded and analyzed.  In the Morris water maze test, the time spent to find the target was recorded during the 7-day training period, with a 90 s cut-off time. And in the last day of 5-min free swimming, the distance travelled and time spent in each quadrant of the pool was recorded and analyzed. | - |
| **Statistical methods** | 13 | Results are expressed as the mean ± SEM. Unpaired t-tests were used to compare differences between groups in various experiments. The criterion for statistical significance was defined as p<0.05. | 8 |
| **RESULTS** | | |  |
| **Baseline data** | 14 | During our study, all the mice were healthy and housed under specific-pathogen-free condition. For the average weight of mice used in food intake measurement, the Rotarod test and the open field test, please see the results of body weight measurement. The weight of WT and CD146^ns-ko^ mice used in the Morris water maze test is tabulated as follows.   | experiment | **The Morris water maze test** | | | | | --- | --- | --- | --- | --- | | group | WT | | CD146^ns-ko^ | | | gender | male | female | male | female | | average weight (g) | 23.68 ± 0.8623 | 17.76 ± 0.4259 | 20.96 ± 0.6110 | 17.15 ± 0.2277 | | - |
| **Numbers analysed** | 15 | For the number of mice in each group of each experiment, please see the sample size section. In all the experiments, data collected from all the mice were included in the analysis. | - |
| **Outcomes and estimation** | 16 | The results of each experiment are tabulated as follows (KO represents for CD146^ns-ko^)  .   | experiment | **The length of the olfactory bulb** | | | | --- | --- | --- | --- | | group | WT | KO | | | normal | abnormal | | ratio | 12/12 | 10/15 | 5/15 | | length (mm) | 2.576 ± 0.084 | 2.658 ± 0.129 | 1.648 ± 0.115 |  | experiment | **The body weight measurement** | | | | | | | | | | | | | --- | --- | --- | --- | --- | --- | --- | --- | --- | --- | --- | --- | --- | | age | 1-month | | | | 3-month | | | | 6-month | | | | | gender | male | | female | | male | | female | | male | | female | | | group | WT | KO | WT | KO | WT | KO | WT | KO | WT | KO | WT | KO | | body weight (g) | 16.79 ± 0.7249 | 14.69 ± 0.7249 | 13.76 ± 1.489 | 11.74 ± 1.209 | 25.72 ± 0.5468 | 20.52 ± 1.136 | 22.86 ± 0.6408 | 18.63 ± 1.225 | 45.30 ± 3.064 | 36.70 ± 1.602 | 37.08 ± 1.620 | 29.20 ± 2.554 |  | experiment | **The food intake measurement** | | | | | | | | | | | | | | | | | --- | --- | --- | --- | --- | --- | --- | --- | --- | --- | --- | --- | --- | --- | --- | --- | --- | | gender | male | | | | | | | | female | | | | | | | | | time | Week 1 | | Week 2 | | Week 3 | | Week 4 | | Week 1 | | Week 2 | | Week 3 | | Week 4 | | | group | WT | KO | WT | KO | WT | KO | WT | KO | WT | KO | WT | KO | WT | KO | WT | KO | | Daily food intake (g) | 4.14 ± 0.5777 | 3.01 ± 0.09458 | 3.59 ± 0.3077 | 2.52 ± 0.2007 | 3.91 ± 0.4025 | 2.86 ± 0.1687 | 4.77 ± 0.4297 | 3.65 ± 0.5214 | 3.25 ± 0.1381 | 2.40 ± 0.1757 | 3.38 ± 0.3572 | 2.50 ± 0.2015 | 3.89 ± 0.6154 | 3.13 ± 0.2484 | 3.72 ± 0.3701 | 2.66 ± 0.2563 |  | experiment | **The Rotarod test** | | | | | --- | --- | --- | --- | --- | | age | 1-month | | 3-month | | | group | WT | KO | WT | KO | | falling latency (s) | 19.13 ± 1.876 | 20.12 ± 1.604 | 15.55 ± 2.163 | 16.81 ± 1.430 |  | experiment | **The open field test** | | | | | | | --- | --- | --- | --- | --- | --- | --- | | age | 1-month | | 3-month | | 6-month | | | group | WT | KO | WT | KO | WT | KO | | distance travelled (mm) | 7632 ± 777.2 | 7232 ± 698.3 | 9331 ± 1013 | 6258 ± 438.2 | 6283 ± 1112 | 4606 ± 682.4 | | rest time (s) | 56.05 ± 7.391 | 63.09 ± 9.376 | 38.95 ± 6.888 | 72.84 ± 7.996 | 74.12 ± 9.302 | 107.3 ± 12.71 | | maximum speed (mm/s) | 158.2 ± 7.663 | 146.3 ± 7.209 | 206.5 ± 13.59 | 168.9 ± 10.63 | 167.3 ± 11.39 | 134.7 ± 10.49 |  | experiment | **The Morris water maze test (Training period)** | | | | | | | | | | | | | | | --- | --- | --- | --- | --- | --- | --- | --- | --- | --- | --- | --- | --- | --- | --- | | training time | Day 1 | | Day 2 | | Day 3 | | Day 4 | | Day 5 | | Day 6 | | Day 7 | | | group | WT | KO | WT | KO | WT | KO | WT | KO | WT | KO | WT | KO | WT | KO | | escape latency (s) | 62.93± 8.069 | 50.32± 6.996 | 60.49± 8.852 | 37.45 ± 6.390 | 56.16 ± 8.283 | 34.20 ± 6.554 | 58.99 ± 9.273 | 30.73 ± 5.726 | 44.33 ± 9.559 | 25.11 ± 4.619 | 59.12 ± 11.24 | 28.28 ± 5.492 | 40.41 ± 6.474 | 17.73 ± 4.542 |  | experiment | **The Morris water maze test (Test day)** | | | | | | | | | --- | --- | --- | --- | --- | --- | --- | --- | --- | | group | WT | | | | KO | | | | | distance travelled (mm) | 5907 ± 694.8 | | | | 6149 ± 650.3 | | | | | p value | 0.8019 | | | | | | | | | quadrant | SW (trained quadrant) | NW | NE | SE | SW (trained quadrant) | NW | NE | SE | | time in each quadrant (%) | 36.86 ± 3.068 | 24.82 ± 4.674 | 16.52 ± 4.314 | 21.79 ± 4.880 | 30.50 ± 1.388 | 23.99 ± 3.357 | 16.89 ± 3.156 | 28.63 ± 4.387 | | 9-12 |
| **Adverse events** | 17 | We report no adverse events during all our experiments. | - |
| **DISCUSSION** | | |  |
| **Interpretation/scientific implications** | 18 | The function of CD146 in the development and maintenance of the CNS has not been studied in great detail, despite its wide distribution in the CNS and its involvement in several neural processes. Whilst previous studies have investigated the function of CD146 *in vitro* and *ex vivo*, here, we provide the first *in vivo* evidence for an essential role of CD146 in mammlian nervous system development and maintenance. Using a CD146 tissue-specific knockout mouse system, we showed that lack of CD146 expression in the nervous system resulted in specific and significant physical and psychological deficiencies. First, CD146^ns-ko^ mice exhibited reduced food intake and were smaller compared with WT littermates. Second, in the open field test, locomotor activity in CD146^ns-ko^ mice was markedly decreased in an age-related manner. Third, spatial learning and memory capacity was impaired in these mice in the Morris water maze test. Interestingly, these deficiencies did not occur simultaneously, but at different stages of life, showing that CD146 is crucial both in the early development and maintenance of the nervous system in the adult mouse. Moreover, the deficiencies observed were in highly specific processes and did not result from a broader impairment of the CNS, as indicated by results of the Rotarod test, in which CD146^ns-ko^ mice exhibited normal balance comparable with WT litter mates.  Deletion of CD146 in the nervous system of mice disrupted appetite and food consumption, the only path of energy intake in animals. To our knowledge, this deficiency has not been reported in other CAM knockout mice. Appetite regulation is a poorly understood and complex process involving the gastrointestinal tract, hormones and the nervous system. Disrupted appetite causes anorexia and malnutrition in humans. Our results provide new insights into appetite regulation, suggesting that CAMs may take part in this process.  In the mammalian brain, the hippocampus is the most important area that responsible for learning and memory. Intriguingly, hippocampal neurons show strong CD146 surface expression, yet we were unable to identify any structural abnormalities in this area in mice lacking CD146. Even though the exact molecular mechanisms remain unclear, we reason that a compensatory mechanism during CNS development might be responsible for the partial restoration of the lack of CD146 in our CD146^ns-ko^ mice, resulting in little or no structure change but memory impairment. Unexpectedly, some CD146^ns-ko^ mice had very small olfactory bulbs, while CD146 expression was not detectable in olfactory bulbs in adult mice. We propose that, similar to NCAM, CD146 might be expressed on neural stem cells during the early stages of development and facilitates the migration of these cells to the olfactory bulb. Thus elimination of CD146 affected neural stem cell migration and resulted in not fully developed olfactory bulbs. Interestingly, this only happened in a small proportion of mice, suggesting an auxiliary function for CD146 and the existence of redundant mechanisms, thus giving rise to a random effect, rather than being deterministic.  CAMs are known to be of particular importance during the development of the nervous system. For instance, NCAM and L1 participate in neuron survival and migration as well as axon growth, fasciculation and path-finding, and knockout of these molecules in rodents leads to a malfunction of the nervous system. The phenotype of CD146^ns-ko^ mice resembles that of NCAM and L1 knockout mice. NCAM knockout mice have small olfactory bulbs and defective spatial learning, however, no decrease in their locomotor activity has been observed. In contrast, decreased body weight, defective locomotor activity and spatial learning have been observed in L1 knockout mice with no abnormalities in olfactory bulb development. Since both the gene sequences and knockout phenotypes in these mice are similar, we propose that CD146 plays a similar role to NCAM and L1, possibly resulting in redundancies in signal pathways that are important for nervous system development. Our study not only demonstrates an important role of CD146, but also provides further proof that CAMs are essential in the development of the nervous system. | 12-15 |
| **Generalisability/ translation** | 19 | Inactivation of CAMs in the nervous system of rodents resembles some gene mutations observed in humans, which are linked to brain disorders and psychological diseases. The possibility of an involvement of CD146 mutations in human psychological diseases such as anorexia and mental retardation warrants further investigation of this essential neuronal cell surface CAM. | 15 |
| **Funding** | 20 | This work was supported by grants from the National Natural Science Foundation of China (Nos. 91029732 and 81272409), National Basic Research Program of China (973 Program; 2009CB521704), "Strategic Priority Research Program" of the Chinese Academy of Sciences, Stem Cell and Regenerative Medicine Research (XDA01040409), and the National Important Science and Technology Specific Projects (2012ZX10002009-016). | - |
